# Facial erythema after the treatment of dupilumab in SLE patient

**DOI:** 10.1186/s13223-020-00458-6

**Published:** 2020-07-03

**Authors:** Dong Hyek Jang, Jae In Lee, Joo Yoon Bae, Hye Jung Jung, Mi Yeon Park, Jiyoung Ahn

**Affiliations:** Department of Dermatology, National Medical Center, 245 Eulji-ro, Jung-gu, Seoul, 04564 Korea

**Keywords:** Atopic dermatitis, Dupilumab, Facial erythema, SLE

## Abstract

**Background:**

Dupilumab is a receptor antagonist binding to the alpha subunit of the interleukin-4 receptor. Through binding to it, dupilumab inhibits signaling of both IL-4 and IL-13, the representative Th2 biomarkers. Recently, in addition to the treatment effects for atopic dermatitis (AD), there is an emerging adverse event as facial erythema.

**Case presentation:**

A twenty-seven-year-old female patient developed erythema and desquamation on the face and neck after dupilumab administration. She had AD on her arms, legs, and trunk before the treatment but there was no atopic clinical feature in her face and neck. With the treatment of dupilumab, her skin lesions of the body have improved from the beginning of the treatment. In the patch test, including dupilumab, there was no specific finding other than the 1+ response to neomycin on day 2. In the intradermal test to dupilumab, a positive result was observed 15 min later, but negative both days 1 and 2. The blood examination showed an elevation of both ANA as 1:80 and anti-phospholipid antibodies (Anti-cardiolipin IgM, IgG, and Anti- beta 2 GPI IgG). She was diagnosed with Systemic lupus erythematosus (SLE) based on diagnostic criteria by a rheumatologist.

**Conclusion:**

Dupilumab is an emerging therapeutic agent for AD, and treatment cases are increasing in Korea. However, there are several adverse events during the treatment of dupilumab. Herein, we report the unexpected adverse event during the treatment of dupilumab in SLE patients.

## Background

Atopic dermatitis (AD) is a chronic inflammatory disease accompanied by itching, which often occurs at any age and gradually improves but sometimes worsens. Mainly, adult atopic dermatitis affects the quality of life because the degree of dermatitis is so severe that healthy daily life cannot be guaranteed. Therefore, there has been much effort in developing treatments for adult atopic dermatitis. Currently, dupilumab (an anti-IL-4R alpha monoclonal antibody [1]) is already an excellent treatment option. Although dupilumab has been very effective in clinical trials and real-world data, there are some treatment-related adverse events. Among them, facial erythema after dupilumab treatment is an emerging adverse event. It has recently been reported frequently at home and abroad [2–4].

## Case presentation

**Fig. 1:**
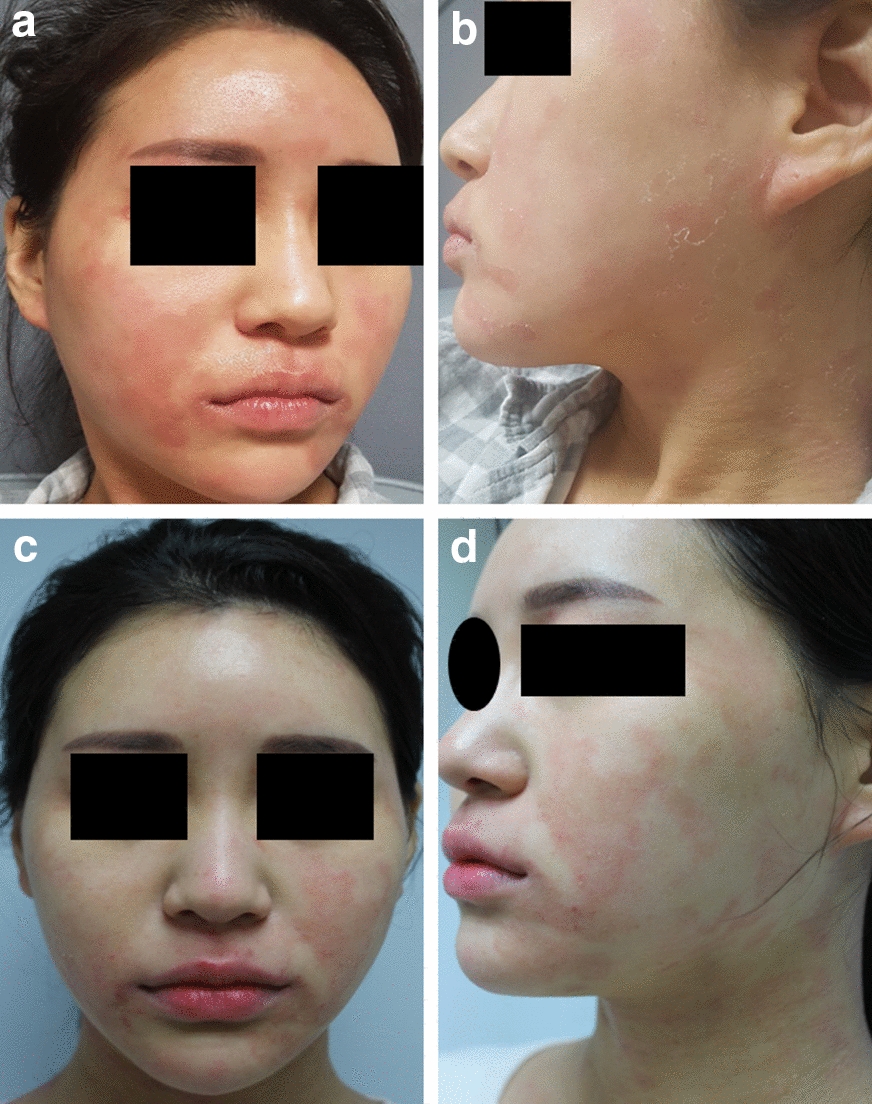
**a**, **b** 2 days after fourth injection (**c, d**) 6 days after fourth injection

**Fig. 2:**
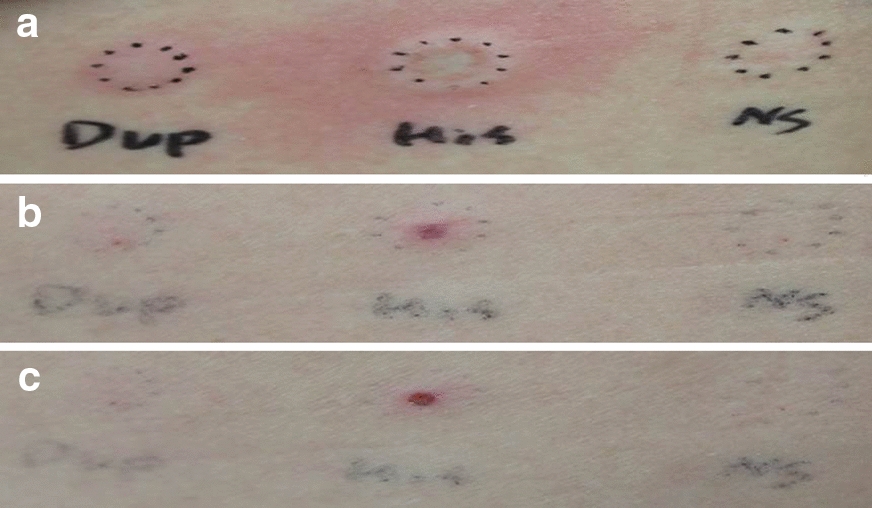
Interdermal test (**a**) 15 min, Dupilumab (3+, same wheal size as histamine) (**b**) 1 day, Dupilumab (−) (**c**) 2 day, Dupilumab (−)

A twenty-seven-year-old female patient developed erythema and desquamation on the face and neck after dupilumab treatment (nasolabial fold was spared). She received first dupilumab treatment at another tertiary hospital with her initial EASI (Eczema Area and Severity Index) score of 16.5 and transferred to our dermatologic clinic. She did not have the symptoms before dupilumab treatment, and they gradually improved after a week (Fig. 1). The same symptoms repeated every time the treatment was administered. Previously she used topical steroid and topical calcineurin inhibitor, but she never used systemic steroid or immunosuppressant such as cyclosporine. She had AD lesions on her arms, legs, and trunk before the treatment, but her face and neck showed no AD lesion at that time. Dupilumab was administered every 2 weeks, and atopic lesions of the body improved from the beginning of the treatment (showed an improvement in the EASI score to 5.5). To identify the cause of the facial erythema after dupilumab treatment, we performed several tests. The patch test, including dupilumab showed negative response except for the 1+ response to neomycin on day 2, which may be thought of as non-specific finding. In the intradermal test (ID test) to dupilumab, a positive result was observed after 15 min (the wheal size of dupilumab was the same as that of histamine). However, negative results were observed on both days 1 and 2 (Fig. 2). Although the ID test showed an immediate reaction, the patient had not experienced any immediate reactions after any dupilumab treatments. Moreover, a delayed reaction was not observed in the patch test or ID test on days 1 and 2, suggesting that the possibility of any hypersensitivity to dupilumab was highly unlikely.

The blood examination results showed elevation of both ANA to 1:80 (speckled type) consistent with the previous result as ANA as 1:320 and anti-phospholipid antibodies (Anti-cardiolipin IgM, IgG, and Anti- beta 2 GPI IgG). However, other autoantibodies were negative, including anti-histone antibody. We consulted a rheumatologist, under the suspicion of Anti-phospholipid antibody syndrome. Also, intermittent leukopenia, the elevation of anti-phospholipid antibodies after three months, and low complement level (C3) were identified. She complained of intermittent pain of both knee joints showing the joint space narrowing on knee x-ray. We recommended a skin biopsy to confirm whether the facial erythema is cutaneous lupus or not, but she refused it. According to SLICC (Systemic Lupus Erythematosus International Collaborating Clinics) criteria [5], she was diagnosed with SLE (Arthritis and Leukopenia in clinical criteria and ANA, Anti-phospholipid antibody and Low complement in immunologic criteria). She took a low dose of aspirin and hydroxychloroquine for SLE. She maintained the dupilumab treatment for AD as her EASI score has improved with the treatment.

### Discussion and conclusions

Erythema of the face after treatment with dupilumab can be caused by (1) worsening of the existing atopic dermatitis lesions, (2) withdrawal of systemic steroid [6] or immuno-suppressant, (3) concomitant allergic contact dermatitis (ACD) [7, 8] or other eczematous skin diseases, (4) a seborrheic dermatitis-like reaction to facial Malassezia species [3] and (5) adverse effects of dupilumab. Our patient did not have any AD lesions on the face at first. Besides, the facial and neck lesions were different from those of flare-up of atopic dermatitis. After the administration of dupilumab, the remaining lesions of the body improved. Nevertheless, the face and neck had worsened with erythema and desquamation. The patient used only topical agents for maintenance during the treatment of dupilumab, so the rebound effect due to the withdrawal of systemic agents can be excluded. We can also exclude allergic contact eczema due to the result of no delayed response in the patch test and intradermal test. Moreover, there have been some cases of ACD treated with dupilumab [8], implying that our patient’s erythema might not have been a lesion of ACD. The following excluded the possibility of seborrheic dermatitis-like reaction: the lesion did not occur in seborrheic areas and showed improvement without topical ketoconazole. The erythema and desquamation after treatment with dupilumab were not reported in previous clinical trials [1]. These reasons caused us to consider another cause of the erythema of the face after treatment with dupilumab. Besides, in one case reported in the United States, facial erythema was reported after the administration of dupilumab [4]. The elevation of ANA was reported in that patient.

This case is the first case of facial erythema after treatment with dupilumab for AD accompanied with SLE. Both AD and SLE are immune diseases involving interactions between genes and the environment [9, 10]. A previous study reported epidemiological correlations and a substantial pathophysiological relationship between AD and SLE [10]. In patients with AD, various autoantibodies have been identified [11–13]. Significant associations between AD and SLE have been reported, implying a shared autoimmune mechanism [11].

Recently, there was a case that showed an erythrodermic psoriasis in a patient treated with dupilumab [14]. It is thought that the opposing shift toward Th1 and Th17 cells by the blockade in the Th2 inflammatory cascades causes a psoriasiform eruption. Guimarães et al. suggested that Th1 and Th17 interaction with lowered Th2 activity is essential in SLE [15]. Therefore, we assumed that the immune shift toward Th1 and Th17 by dupilumab resulted in the facial erythema as a form of cutaneous lupus in our patient.

However, there is a possibility that the facial erythema is the skin manifestation of drug-induced lupus (DIL) caused by dupilumab. Although DIL to an existing commonly known drug-like procainamide mostly has positive anti-histone antibody, a newly developed drug-like biologic agent often shows negative anti-histone antibody [16]. One of the main ways to diagnose DIL is improvement after discontinuing suspicious agents. Unfortunately, our patient did not want to discontinue the treatment of dupilumab. Therefore, we can’t entirely exclude the possibility of DIL. Further studies on erythema after treatment with dupilumab in patients with SLE will be necessary in the future.

As treatment with dupilumab has increased, various treatment-related cases are expected to be reported. We believe that our case will help understand one of the causes of facial erythema after dupilumab treatment.

## Data Availability

Not applicable.
